# Drivers to Stay or to Leave: Perceptions of the Nursing Work Environment Across Diverse Healthcare Sectors

**DOI:** 10.1155/jonm/5544729

**Published:** 2026-03-19

**Authors:** Denise M. Connelly, Nicole A. Guitar, Kristin Prentice, Melissa E. Hay

**Affiliations:** ^1^ School of Physical Therapy, Western University, London, Ontario, Canada, uwo.ca

**Keywords:** culture, home care services, hospitals, long-term care, nurses, nursing, surveys and questionnaires

## Abstract

**Background:**

Long‐term care and home and community care sectors are not perceived as workplaces of choice for nurses.

**Aim:**

To describe (1) what creates a desirable work environment in long‐term care, home and community care, and hospital sectors for nurses; (2) what motivates nurses to work in these sectors; and (3) what influences nurses to leave these sectors when they do.

**Design:**

An open, cross‐sectional online survey.

**Methods:**

Registered Practical Nurses and Registered Nurses across Ontario were recruited broadly to rate 14 statements in a survey regarding their beliefs about three healthcare sectors (i.e., long‐term care, home and community, and hospital sector) and 20 statements regarding their perceptions of motivation, and attrition‐related factors, to work in these sectors.

**Results:**

Data from Registered Practical Nurses (*N* = 264) and Registered Nurses (*N* = 109) were analyzed (total *N* = 373, completion rate = 90.7%). Nurses report that they leave work in long‐term care and/or home and community care because they are seeking better work–life balance, an increase in pay, and because they do not feel valued or appreciated by their employer. While nurses agree that their schooling has prepared them well to work in these sectors, they also agree that they would be more motivated to work within these sectors if they were able to determine their own availability.

**Conclusion:**

Financial and nonfinancial influences were reported within the motivating and push factors for nurses to work in these sectors. The reported beliefs of nurses enforced the stigma attached to working as a nurse within these two sectors and provided novel insight into the system and organizational challenges for nurse workforce recruitment and retention.

**Summary**
• **Implications for Nursing Management**
◦ These results inform how management can leverage motivating and push factors within these strained sectors of the healthcare system to enhance workforce recruitment and retention.
• **Reporting Method**
◦ The Checklist for Reporting the Results of Internet E‐Surveys (CHERRIES) was used.
• What Does This Paper Contribute to the Wider Global Clinical Community?
◦ This survey offers novel insights at a regional level about motivational factors for nurses to work within the long‐term care and home and community care sectors, as well as push factors contributing to the efflux of nurses from these two sectors to other employment settings.
◦ These findings may inform recruitment and retention strategies to address high workloads, job enlargement, and widespread burnout contributing to nursing shortages in long‐term care and home and community care sectors.
◦ This research adds to the extant body of literature invoking the system and organizations to enhance staff and management resources and to provide financial/nonfinancial remuneration to address workforce attrition within long‐term care and home care settings.
• **Patient or Public Contribution**
◦ Questions were developed in co‐design with a Nursing Knowledge Advisory Group (NKAG) formed for the purpose of advising on a series of nursing research studies. The NKAG is a group comprised of 23 nurse educators, students, researchers, professionals, and community stakeholders involved in nursing education, policy, and practice. This group also pilot tested the survey prior to its’ release.

## 1. Introduction

Within the context of rebuilding the nursing workforce in the long‐term care (LTC) and home and community care (HCC) sectors, the assumptions that these are not workplaces of preference for nurses [1–3] need to be examined. The literature reports long‐held preconceived notions by nurses that working with older adults (i.e., people ≥ 65 years of age), the majority client group in LTC and HCC sectors [4], is uncomplicated and “slow” ([5], p. 284). Currently, LTC presents as a stressful work environment secondary to increasing resident medical complexities, infrastructural deficiencies, a lack of resources, and chronically insufficient staffing levels [6]. In HCC, the work environment holds unique challenges for nurses working in client spaces that are not designed to facilitate their clinical duties [7]. These stressful work environments were exacerbated by the coronavirus disease 2019 (COVID‐19) pandemic [8–10]. With an aging population, demand for community‐based care is increasing, placing pressure on an already stressed nursing workforce [11–13].

Although older adults comprise an increasingly large portion of patient caseloads in hospital settings [14, 15], caring for older adults in LTC and HCC environments is perceived as undesirable work by nurses, with multiple “push” factors (e.g., poor public image of nursing, workplace pressures) out of the profession [2, 5]. In the past, nurses have expressed greater anxiety about working with older adults when compared to people of other age groups, and have qualified working with older adults as “low status” [3, 16]. For decades, some nurses have expressed “negative attitudes [that] reflect ageist stereotypes and knowledge deficits that significantly influence [nurses’] practice and older patients’ quality of care” ([17], p. 62 [18]).

To frame the aim of this paper, that is, to understand what creates a desirable work environment in LTC, HCC, and hospital sectors, and what motivates nurses to work in or to leave these healthcare sectors, the existing literature from nurses working in hospitals provides context. Previous research exploring nurses’ desired work conditions in hospitals suggests there are eight domains important to nurses: established wages that increase with inflation, supply of personnel and adequate staffing levels, maintenance of scope of practice, respect for flexibility of work hours and work–life balance, resources for students and nurses with families, ongoing educational opportunities, supporting nurse self‐esteem, and other incentives like allowing participation by nurses in hospital policy decisions [19]. Notably, nurses’ desired work conditions in the hospital sector have been thoroughly studied, but little research on desired workplace factors were found for the LTC and HCC sectors [20–26]. Research in hospitals from the 2000s suggested that low pay and benefits were primary factors that led to nurse job dissatisfaction and nurse turnover, demonstrating that wage parity is an ongoing issue [27]. However, supervisors, job characteristics, and management styles, as elements of a practice environment, are key factors associated with nurses having “a positive practice milieu,” which is known to improve nurses’ satisfaction and minimize turnover ([27], p. 525; [28–30]).

Little is known about what motivates nurses to work within sectors like LTC and HCC, and what factors contribute to leaving work in these sectors. Given the current climate of nurse shortages due to excessive workloads, increasing job demands, and burnout, understanding the current perceptions that nurses hold about working in different workplace sectors is paramount to promoting recruitment and retention in currently strained global healthcare systems [31–35]. Therefore, there is a gap in our understanding of what motivates nurses to stay and leave and how to address these motivating and demotivating factors with solutions for nurses working within the environments of the LTC and HCC sectors.

## 2. Aim

The aim of this study is to describe (1) what creates a desirable work environment in LTC, HCC, and hospital sectors for nurses; (2) what motivates nurses to work in LTC and/or HCC; and (3) what influences nurses to leave these LTC and/or HCC.

## 3. Materials and Methods

### 3.1. Ethics

Ethical approval for the study was obtained from the University of Western Ontario’s institutional ethics review board (review reference number: 2023–121762‐7488) on January 11, 2023.

### 3.2. Survey Design and Development

Qualtrics XM (Provo, UT) online software was used to conduct a voluntary open online survey, which was accessible for 8 months via computer or smartphone between March 27, 2023, and November 20, 2023. The survey was only available in English. No incentives or compensation was provided to respondents. Survey content and organization was developed iteratively among the research team and a Nursing Knowledge Advisory Group (NKAG) using The Tailored Design Method [36]. The NKAG was formed for the purpose of advising on which survey items, generated from the literature, to use in this nursing research study. The NKAG was a group of 23 composed of nurse educators, nurse students, researchers, professional nurse association members, and community stakeholders involved in nursing education, policy, and practice. Together, the authors and the NKAG discussed and determined what questions to include in the online survey. Several members from the NKAG pilot tested the functionality of the survey prior to its release.

The survey included multiple‐choice and Likert‐scale questions about demographic characteristics (e.g., age, gender, marital status, education level, work experience, income) and asked questions about nurses’ work in LTC, HCC, and/or hospital sectors. Respondents were asked to rate factors identified by the research team and NKAG that contributed to people leaving their work (10 items) in either LTC or HCC; their beliefs (14 items) about working in LTC, HCC, or hospital settings; and features that would motivate them to work (20 items) in LTC or HCC. All items were rated on a seven‐point Likert scale from 1—*strongly disagree* to 7—*strongly agree*. No standardized outcome measures were included within the survey.

The survey contained 13 pages in total, and the number of questions per page ranged from one to five. The landing page of the survey contained a letter of information (LOI) and asked all respondents to indicate their informed consent prior to allowing respondents to answer other questions through a series of tick boxes. Respondents were informed that all data collected would be anonymous. A total of 46 questions were presented to respondents; however, some of those questions were matrix tables containing up to 20 statements for which a respondent was asked to provide a response. Respondents were given the option to navigate backwards in the survey, to skip questions, or not provide a response to a question. Adaptive questioning was used to provide specific questions based on whether a respondent reported leaving work in a sector (e.g., if they left work in LTC, they were asked additional questions about the circumstances). There was no time limit for completion of the survey, item randomization was not used, and no consistency check took place.

### 3.3. Setting, Sample, and Recruitment

LTC settings include places like nursing homes, rehabilitation and skilled nursing facilities, and inpatient behavioral health facilities. They are residential homes that provide ongoing care to patients whose care needs cannot be met in the community [37]. In contrast, HCC is provided in settings like clients’ homes, schools, and communities [38]. Hospital settings include any institution, building, or other premises or place that is established for the purposes of the treatment of patients, sometimes called “acute care” [39]. In both LTC and HCC settings in Ontario, Canada, for example, Registered Practical Nurses (RPNs) and Registered Nurses (RNs) comprise most of the registered workforce providing direct care [40]. Across the globe, other nurse titles that have a similar scope to the Ontario RPNs in this research include Licensed Practical Nurses in other Canadian provinces [40, 42, 43], Enrolled Nurses in Australia and New Zealand [44, 45], and Assistant Practitioners, Associate Degree and Associate Nurses in the United Kingdom [46, 47].

Any currently practicing RPN or RN working in any healthcare setting across the province of Ontario who could read and write in English was invited to participate. Respondents were recruited through ongoing free advertisements with the Registered Practical Nurses Association of Ontario (WeRPN; sent to ∼16,000 subscribers). Other professional associations we approached declined the opportunity to share our recruitment advertisement. A paid advertisement posted to the Hospital News website homepage was used for recruitment and attracted approximately 5000 views over the course of the 30 days that it was posted. WeRPN and Hospital News provided a direct link to the survey where potential participants landed on the page with the LOI.

### 3.4. Data Management and Statistical Analyses

All data were exported from Qualtrics into Microsoft Excel software. Quantitative data analyses were completed using SPSS Version 29 (IBM). *A priori*, the authors decided to exclude surveys that were < 80% complete from descriptive statistic calculations. Descriptive statistics were used to analyze responses, and Mann–Whitney *U* tests were used to determine if there were significant differences in the responses of RPNs when compared to RNs. A *p* < 0.05 was used.

All responses were anonymous and securely stored on a firewall‐protected computer. Encryption technology and restricted access authorizations within Qualtrics were used to prevent the collection of any respondents’ system data (e.g., IP addresses, cookies, and geographic locations). No other log file analyses were used, and no direct contact was made with potential respondents. It was not possible to calculate a participation rate secondary to the use of nonprobabilistic sampling, that is, it could not be determined how many eligible people were exposed to the invitation to participate [48, 49]; therefore, a participation rate was not calculated, but a completion rate of how many nurses completed the survey that consented was calculated.

### 3.5. Research Reporting Checklist

The Checklist for Reporting Results of Internet E‐Surveys (CHERRIES) was used in the writing of this manuscript [50].

## 4. Results

A total of 411 nurses consented to completing the survey; however, 31 were < 80% complete and 7 were completed in < 3 min (i.e., a time frame deemed unfeasible by the research team *a posteriori*) and were excluded. Therefore, a total of 373 surveys, completed by 264 RPNs and 109 RNs, were included in the analysis; see Table 1 for respondent demographics (completion rate = 90.7%). For the 373 surveys included in the analysis, the median and mode times for survey completion were 12.9 and 13.0 min, respectively. Table 2 showcases the characteristics of the LTC and HCC places of respondents’ work. Table 3 highlights the work experiences of respondents in LTC and HCC.

**TABLE 1:** Frequency distribution table of respondent characteristics (*N* = 373).

Characteristic	RPN (*N* = 264) (%)	RN (*N* = 109) (%)
*Gender*		
Woman	92.0	88.1
Man	6.4	8.3
Nonbinary	1.1	0.0
Prefer not to say	0.0	1.8
Genderqueer	0.0	0.9

*Marital status*		
Married/common law	70.5	62.4
Single	20.8	24.8
Separated/divorced	6.4	8.3
Prefer not to say	1.1	2.8
Widowed	0.8	0.9

*Education level*		
College diploma	86.0	23.9
Undergraduate degree	9.1	45.9
Master’s degree	1.9	15.6
Graduate degree	1.5	12.8
High school diploma	1.1	0.0
Doctoral degree	0.0	0.9

*Income in the previous year*		
≥ $60,000	22.7	74.3
$50,000–59,999	48.5	11.0
$40,000–49,999	13.6	3.7
$30,000–39,999	5.3	5.5
$10,000–19,999	3.0	2.8
$20,000–29,999	3.8	0.0
< $5000	1.5	0.9
$5000–9999	0.4	0.0

*Current sector of nursing employment*		
Hospital	49.2	69.7
LTC	22.3	7.3
HCC	17.8	12.8
Other	9.8	10.1

*Employment status*		
Full‐time	68.6	67.9
Part‐time (1 or more positions)	18.2	17.4
In a combination of the above	8.7	9.2
Casually	3.4	5.5

*Ontario health region*		
Southwest	17.8	38.5
Unsure of my region	16.3	5.5
Central East	11.7	8.3
Waterloo Wellington	8.7	0.9
North Simcoe Muskoka	7.2	4.6
Hamilton Niagara Haldimand Brant	6.4	5.5
Toronto Central	5.3	11.0
Southeast	5.3	1.8
Mississauga Halton	4.9	3.7
Champlain	4.2	6.4
Northeast	4.2	3.7
Erie St. Clair	3.4	1.8
Central	1.5	4.6
Northwest	1.1	1.8
Central west	1.1	0.9

*Note:* Data are presented as frequencies in the order of highest to lowest for the first column—RPNs; however, not all respondents may have answered all questions, meaning a sum of 100% for each variable may not be present.

Abbreviations: HCC = home and community care, LTC = long‐term care, RN = Registered Nurse, RPN = Registered Practical Nurse.

**TABLE 2:** Sector‐specific demographic characteristics for nurses who indicated they have worked in LTC and HCC.

	**LTC**		**HCC**	

*Work experience in listed sector?*	RPN (*N* = 262) (%)	RN (*N* = 108) (%)	RPN (*N* = 261) (%)	RN (*N* = 109) (%)
Yes	77.9	50.9	45	40.4
No	22.1	49.1	54.4	59.6

*Working currently in sector?*				
Yes	23.3	12.0	13.4	11.9

*Facility size* ^a^				
Small (1–96 residents)	16.4	15.7	—	—
Medium (97–160 residents)	30.5	18.5	—	—
Large (≥ 161 residents)	28.6	15.7	—	—

*Number of residents assigned per nurse*				
≤ 10	6.5	5.6	23.8	22.9
11–20	9.9	9.3	16.1	12.8
21–30	19.8	13.0	3.1	0.0
≥ 31	40.8	22.2	1.9	2.8

*Funding structure at place of employment*				
Public	26.7	18.5	14.6	22.0
Private	29.4	14.8	14.2	9.2
Both	13.0	10.2	6.9	6.4
Unsure	8.8	7.4	9.6	2.8

*Note:* Data are presented as frequencies in the order of highest to lowest for the first column—RPNs; however, not all respondents may have answered all questions, meaning a sum of 100% for each variable may not be present.

Abbreviations: HCC = home and community care, LTC = long‐term care, RN = Registered Nurse, RPN = Registered Practical Nurse.

^a^Facility size descriptions were determined in consultation with our Nursing Knowledge Advisory Group.

**TABLE 3:** Nursing experience for RPNs and RNs.

	**Mean**	**SD**	**95% CI**	**Min–max**
			**RPN**	

Years in current position	5.6	6.9	4.8, 6.4	0–41
Years working as a nurse (total)	12.4	10.2	11.2, 13.6	0–47
Hours scheduled weekly (total)	37.6	9.9	36.3, 38.8	0–75

			**RN**	

Years in current position	7.7	7.8	6.1, 9.2	0–36
Years working as a nurse (total)	18.6	12.6	16.2, 21.1	1–47
Hours scheduled weekly (total)	34.8	10.2	32.8, 36.7	6–60

Abbreviations: RN = Registered Nurse, RPN = Registered Practical Nurse.

Mann–Whitney *U* tests were run to determine if there were differences in agreement between RPNs and RNs regarding: (1) beliefs about work in LTC, HCC, and hospital sectors; (2) motivators to work within LTC and HCC sectors; and (3) the push factors for leaving work in LTC and HCC sectors. Distributions were similar, as assessed by visual inspection. Most of the respondents’ ratings were not statistically different between RPNs and RNs using an exact sampling distribution for *U* (see Table 4 for the significant results [51]).

**TABLE 4:** Results of Mann–Whitney *U* tests indicating significant differences between RPNs and RNs ratings of agreeance on the statements.

	*N*	Mean rank	Mean (SD)	*U*	*Z*	*p*
*Beliefs about LTC*						
The pay in LTC is fair	RPN = 242 RN = 102	RPN = 164.81 RN = 190.75	RPN = 2.34 (1.52) RN = 2.25 (1.06)	10,481.00	−2.339	0.019
Working in LTC requires specialized education and training	RPN = 242 RN = 104	RPN = 164.75 RN = 193.85	RPN = 3.94 (1.72) RN = 4.83 (1.70)	10,467.50	−2.520	0.012
Emergency preparedness in this sector would ensure my safety	RPN = 242 RN = 104	RPN = 180.87 RN = 156.35	RPN = 3.81 (2.10) RN = 3.33 (1.92)	10,800.50	−2.118	0.034

*Beliefs about HCC*						
Working in HCC would allow me to use my knowledge/skills to their fullest extent	RPN = 219 RN = 95	RPN = 166.44 RN = 136.88	RPN = 5.28 (1.44) RN = 4.00 (1.86)	13,004.00	−2.696	0.007

*Hospital beliefs*						
My schooling has prepared me well to work in the hospital sector	RPN = 217 RN = 94	RPN = 145.77 RN = 179.62	RPN = 5.00 (1.64) RN = 5.66 (1.17)	7978.50	−3.157	0.002
Work in the hospital sector would facilitate good work–life balance for me	RPN = 217 RN = 94	RPN = 149.47 RN = 171.08	RPN = 3.25 (1.81) RN = 3.70 (1.95)	8781.50	−1.977	0.048

*Would work in LTC if…*						
The pay was higher	RPN = 211 RN = 89	RPN = 158.34 RN = 131.90	RPN = 5.22 (1.70) RN = 4.33 (2.15)	7734.50	−2.473	0.013
There were lighter workloads (e.g., better staff to patient ratios)	RPN = 210 RN = 89	RPN = 156.82 RN = 133.91	RPN = 5.88 (1.45) RN = 4.75 (2.26)	7913.00	−2.215	0.027
The location was close to my home	RPN = 210 RN = 89	RPN = 157.46 RN = 132.40	RPN = 5.19 (1.56) RN = 3.92 (2.39)	7779.00	−2.350	0.019
I was guaranteed full time employment	RPN = 210 RN = 89	RPN = 161.76 RN = 122.25	RPN = 5.00 (1.62) RN = 4.00 (1.65)	6875.50	−3.680	< 0.001
I could determine my own availability	RPN = 210 RN = 89	RPN = 159.70 RN = 127.12	RPN = 5.75 (1.57) RN = 4.67 (1.72)	7308.50	−3.100	0.002
I was guaranteed two consecutive days off per week	RPN = 210 RN = 89	RPN = 164.37 RN = 116.09	RPN = 5.56 (1.66) RN = 3.75 (2.05)	6327.00	−4.563	< 0.001
I was guaranteed access to personal protective equipment (PPE) and other infection prevention resources	RPN = 209 RN = 89	RPN = 157.20 RN = 131.42	RPN = 5.75 (1.41) RN = 4.58 (2.07)	7691.00	−2.463	0.014
There were opportunities for my professional development	RPN = 209 RN = 88	RPN = 159.79 RN = 123.37	RPN = 5.75 (1.27) RN = 4.17 (2.04)	6940.50	−3.43	< 0.001
There were opportunities for my education and training	RPN = 208 RN = 89	RPN = 157.97 RN = 128.03	RPN = 5.75 (1.19) RN = 4.50 (2.11)	7389.50	−2.838	0.005
There were employee benefits that included health and dental care	RPN = 209 RN = 89	RPN = 159.83 RN = 125.24	RPN = 6.19 (1.06) RN = 4.67 (2.39)	7141.00	−3.339	< 0.001
There were rewards and recognitions made available to me	RPN = 209 RN = 89	RPN = 161.26 RN = 121.89	RPN = 5.69 (1.60) RN = 4.25 (2.38)	6843.00	−3.701	< 0.001
There were opportunities to connect with my supervisor(s)	RPN = 209 RN = 89	RPN = 156.83 RN = 132.28	RPN = 3.50 (1.48) RN = 3.50 (1.62)	7767.50	−2.303	0.021

*Would work in HCC if…*						
There were opportunities available for my professional development	RPN = 190 RN = 80	RPN = 143.83 RN = 115.72	RPN = 5.88 (1.13) RN = 5.00 (1.28)	6017.50	−2.810	0.005
There were rewards and recognitions made available to me	RPN = 190 RN = 80	RPN = 142.66 RN = 118.49	RPN = 5.41 (1.58) RN = 4.58 (1.16)	6239.50	−2.385	0.017

*Reasons for leaving LTC*						
My declining physical health because of my work	RPN = 138 RN = 38	RPN = 92.82 RN = 72.83	RPN = 4.50 (1.98) RN = 2.92 (2.20)	2026.50	−2.169	0.030
My declining mental health because of my work	RPN = 138 RN = 36	RPN = 93.17 RN = 65.78	RPN = 5.38 (1.60) RN = 4.00 (2.41)	1702.00	−2.958	0.003
Concerns about my personal safety	RPN = 139 RN = 37	RPN = 92.65 RN = 72.91	RPN = 4.41 (2.21) RN = 3.33 (2.19)	1994.50	−2.120	0.034

*Reasons for leaving HCC*						
Not being valued or appreciated by my employer	RPN = 74 RN = 25	RPN = 49.82 RN = 50.54	RPN = 5.66 (1.72) RN = 4.67 (2.27)	668.00	−2.145	0.032
My declining physical health because of my work	RPN = 74 RN = 25	RPN = 54.45 RN = 36.82	RPN = 4.22 (2.21) RN = 3.08 (2.43)	595.5	−2.688	0.007
My declining mental health because of my work	RPN = 73 RN = 25	RPN = 53.77 RN = 37.02	RPN = 5.09 (1.97) RN = 3.67 (2.54)	600.50	−2.585	0.010

*Note:* Only survey items with *p* < 0.05 are included.

Abbreviations: HCC = home and community care, LTC = long‐term care, RN = Registered Nurse, RPN = Registered Practical Nurse.

### 4.1. Beliefs About the Sectors

#### 4.1.1. The LTC Sector

**FIGURE 1:**
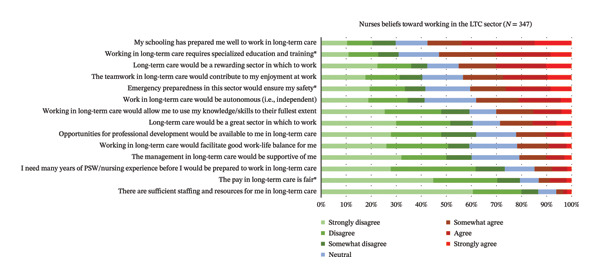
Respondents’ beliefs toward working in the LTC sector (*N* = 347). ^∗^Items where there is a significant difference in the agreeance levels between RPNs and RNs.

The level of agreeance by respondents to 14 statements regarding their beliefs about working in the LTC sector is reported in Figure 1. The three statements with highest agreeance were as follows: (1) *my schooling has prepared me well to work in LTC*; (2) *working in LTC requires specialized education and training*; and (3) *LTC would be a rewarding sector in which to work*. The three least agreed with statements by respondents were as follows: (1) *there are sufficient staffing and resources for me in LTC*; (2) *the pay in LTC is fair*; and (3) *I need many years of Personal Support Worker/nursing experience before I would be prepared to work in LTC*. RPNs agreed significantly more than RNs that the pay in LTC is fair (*p* = 0.019) and that emergency preparedness in the LTC sector would ensure their safety (*p* = 0.034; see Table 4). RNs agree significantly more than RPNs that working in LTC requires specialized education and training (*p* = 0.012).

#### 4.1.2. The HCC Sector

**FIGURE 2:**
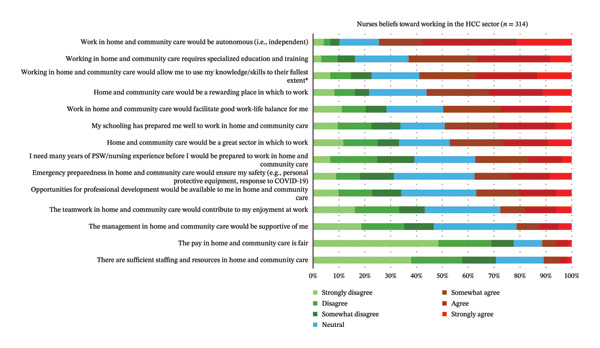
Respondents’ beliefs toward working in the HCC sector (*N* = 314). ^∗^Items where there is a significant difference in the agreeance levels between RPNs and RNs.

The level of agreeance by respondents to 14 statements regarding their beliefs about working in the HCC sector is reported in Figure 2. The top three were as follows: (1) *work in HCC would be autonomous (i.e., independent)*; (2) *working in HCC requires specialized education and training*; and (3) *HCC would allow me to use my knowledge/skills to their fullest extent*. The most disagreed with beliefs for respondents were as follows: (1) *there are sufficient staffing and resources for me in HCC*; (2) *the pay in HCC is fair*; and (3) *the management in HCC would be supportive of me*. RPNs agreed significantly more than RNs that working in HCC would allow them to use their knowledge and skills to their fullest extent (*p* = 0.007; see Table 4).

#### 4.1.3. The Hospital Care Sector

**FIGURE 3:**
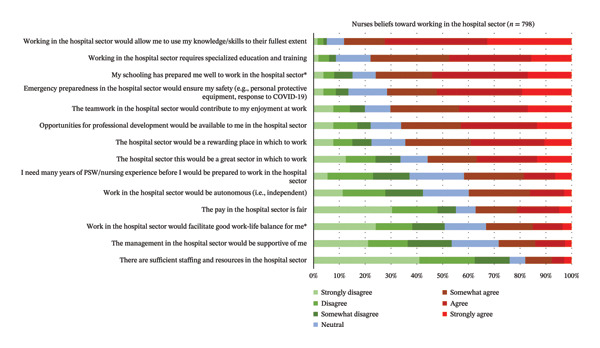
Respondents’ beliefs toward working in the hospital sector (*N* = 310). ^∗^Items where there is a significant difference in the agreeance levels between RPNs and RNs.

The level of agreeance by respondents to 14 statements regarding their beliefs about working in the hospital sector is reported in Figure 3. The top three were as follows: (1) *working in the hospital sector would allow me to use my knowledge/skills to their fullest extent*; (2) *working in the hospital sector requires specialized education and training*; and (3) *my schooling has prepared me well to work in the hospital sector*. The most disagreed with beliefs for respondents were as follows: (1) *there are sufficient staffing and resources for me in the hospital sector*; (2) *the management in the hospital sector would be supportive of me*; and (3) *work in the hospital sector would facilitate good work–life balance for me.* RNs agree significantly more than RPNs that their schooling prepared them well to work in the hospital sector (*p* = 0.002) and that working in the hospital sector would facilitate a good work–life balance (*p* = 0.048; see Table 4).

### 4.2. Motivators to Work Within the Sectors

#### 4.2.1. The LTC Sector

**FIGURE 4:**
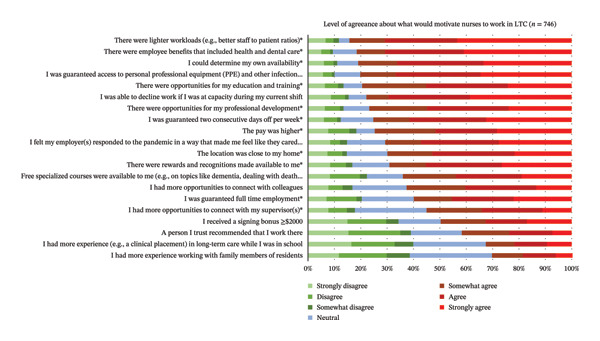
Respondents’ level of agreeance about what would motivate them to work in LTC (*N* = 300). ^∗^Items where there is a significant difference in the agreeance levels between RPNs and RNs.

The most agreed upon factors reported by respondents as aspects that would motivate them to work in LTC are reported in Figure 4. The top three motivators were as follows: (1) *lighter workloads (e.g., better staff to patient ratios)*; (2) *employee benefits that included health and dental care*; and (3) *if I could determine my own availability*. The three factors reported to least motivate respondents to work in the LTC sector were as follows: (1) *needing more experience working with family members of residents;* (2) *needing more experience (e.g., a clinical placement) in LTC while in school*; and (3) *if a trusted person recommended working in LTC*.

RPNs agreed significantly more than RNs that they would work in LTC if the pay was higher (*p* = 0.013), there were lighter workloads (*p* = 0.027), the location was close to their home (*p* = 0.019), they were guaranteed full‐time employment (*p* = <0.001), they could determine their own availability (*p* = 0.002), they were guaranteed two consecutive days off per week (*p* = <0.001), they were guaranteed access to personal protective equipment and other infection prevention resources (*p* = 0.014), there were opportunities for professional development (*p* = <0.001), opportunities for education and training (*p* = 0.005), there were employee benefits that included health and dental (*p* < 0.001), there were rewards and recognitions made available to them (*p* < 0.001), and if there were opportunities to connect with their supervisors (*p* = 0.021; see Table 4).

#### 4.2.2. The HCC Sector

**FIGURE 5:**
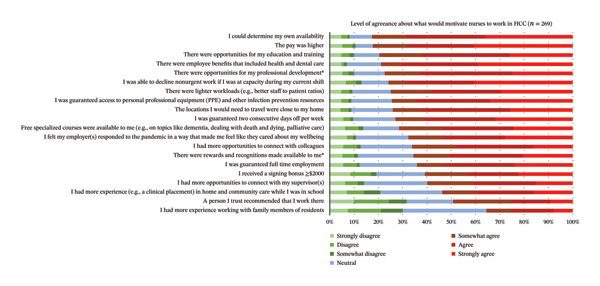
Respondents’ level of agreeance about what would motivate them to work in LTC (*N* = 373). ∗Items where there is a significant difference in the agreeance levels between RPNs and RNs.

The most agreed upon factors reported by respondents as aspects that would motivate them to work in HCC are reported in Figure 5. The top three motivators were as follows: (1) *being able to determine their own availability*; (2) *higher pay*; and (3) *opportunities for education and training*. The three factors reported to least motivate respondents to work in the HCC sector were as follows: (1) *needing more experience working with family members of residents*; (2) *a trusted person recommended working in HCC*; and (3) *needing more experience (e.g., a clinical placement) in HCC while in school*.

RPNs agreed significantly more than RNs that they would work in HCC if there were opportunities for professional development (*p* = 0.005) and if there were rewards and recognitions made available to them (*p* = 0.017; see Table 4).

#### 4.2.3. Reasons for Leaving LTC or HCC Sectors

**FIGURE 6:**
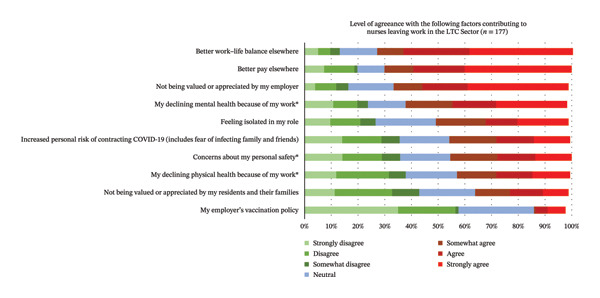
Respondents’ level of agreeance with factors that contributed to them leaving work in LTC (*N* = 177). ^∗^Items where there is a significant difference in the agreeance levels between RPNs and RNs.

Some respondents reported they had worked in LTC (*N* = 177) and/or HCC (*N* = 183) previously but had left their work within that sector. Various factors that participants reported contributing to leaving their work in the LTC sector are reported in Figure 6. The top three reasons were as follows: (1) *better work–life balance elsewhere*; (2) *better pay elsewhere*; and (3) *not being valued or appreciated by my employer*. The three reasons reported to have contributed least to people leaving their work in the LTC sector were as follows: (1) *my employer’s COVID-19 vaccination policy*; (2) *not being valued or appreciated by my residents and their families*; and (3) *my declining physical health because of my work*.

For the nurses who reported having worked in LTC in the past, RPNs agreed significantly more than RNs that reasons they left LTC were their declining physical (*p* = 0.030) and mental health (*p* = 0.003), and concerns for their personal safety (*p* = 0.034; see Table 4).

**FIGURE 7:**
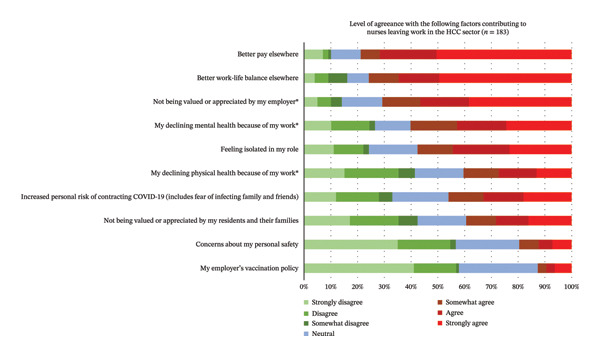
Respondents’ level of agreeance with factors that contributed to them leaving work in HCC (*N* = 183). ^∗^Items where there is a significant difference in the agreeance levels between RPNs and RNs.

Several factors that participants reported as contributing to leaving their work in the HCC sector are reported in Figure 7. The top three reasons were as follows: (1) *better pay elsewhere*; (2) *better work–life balance elsewhere*; and (3) *not being valued or appreciated by my employer*. The three reasons reported to have contributed least to people leaving their work in the HCC sector were as follows: (1) *my employer’s vaccination policy*; (2) *concerns about my personal safety*; and (3) *not being valued or appreciated by my residents and their families*.

For the nurses who reported having worked in HCC in the past, RPNs agreed significantly more than RNs that reasons they left HCC were their declining physical (*p* = 0.007) and mental health (*p* = 0.010) and not feeling valued or appreciated by their employer (*p* = 0.032; see Table 4).

## 5. Discussion

A province‐wide survey investigated: (1) what creates a desirable work environment in LTC, HCC, and hospital sectors for nurses; (2) what motivates nurses to work in LTC and/or HCC; and (3) what factors influence nurses to leave these LTC and/or HCC settings. Respondents’ beliefs about the LTC and HCC sectors differed. Regarding LTC, respondents agreed the most that their schooling prepared them well to work there, but regarding HCC, respondents agreed most that the work would be autonomous. In both sectors, respondents agreed that being able to determine their own availability would make them want to work within those sectors. The results show the reasons nurses reported for leaving their work in LTC and HCC were similar, but for RPNs, they were more related to declining physical and mental health. Both RPNs and RNs agreed most that they left work in LTC and/or HCC because they were seeking better work–life balance, an increase in pay, and because they did not feel valued or appreciated by their employer.

The significant differences between RPN and RN responses may be attributed to the difference in education and training between the two roles. It is possible that RNs believe there needs to be more specialized training for LTC because of the advanced leadership roles and responsibilities they have in the LTC setting. In HCC, RPNs require more autonomy and a diverse skill set [52] than RPN roles in other settings, including LTC, which may lead them to believe that they can use their skills to their fullest extent in that sector. Because the RN role holds more responsibilities than the RPN role, it is also possible that RNs envision using the extent of their skills in other complex settings, such as the Intensive Care Unit. The stigma of working with older adults in LTC and/or HCC could also shape their perception of skills they would use in those settings.

RN roles in Ontario have more career mobility than RPN roles due to their advanced education and broader scope of practice [40]. Although RPNs can work in a variety of settings, like RNs, they do not have all the same role opportunities, which leads many to want to bridge careers to become an RN [53]. Because RNs have more role opportunities, perhaps they have less motivation to work in the LTC and/or HCC settings because they can attain better pay elsewhere. RPNs suggested they would leave working in these sectors due to declining physical and mental health. This could be an indicator that nursing managers in the LTC and HCC settings should focus their attention to how RPNs are taking care of themselves on the job and provide resources as a form of support [54, 55].

Previous research indicates that individuals who enter the healthcare profession of nursing do so because they see an opportunity for caring and fulfilling their vocation—despite chronic struggles with resource management and staff shortages [2, 56]. Nurses tend to score high on measures of empathy and altruism, with a caring nature being a “principal quality of the nurse personality” ([56], p. 1546). Nurses report having a desire to help others and believe that being “caring or compassionate” is the most common personal characteristic that is helpful to have as a nurse ([57], p. 63). Survey research with approximately 2000 nurses in Australia also identified the characteristic of “intrinsic attraction,” or the ability to make a strong contribution to society, to help others and to work closely with people while engaging in mentally interesting, challenging, and exciting work as part of a community and profession is perceived to carry prestige [58]. Intrinsic attraction was noted as contributing more to the decision to become a nurse when compared to the “extrinsic rewards” of the job, such as earning potential, salary, opportunities for promotion and advancement in pleasant working conditions, with flexible hours of work, responsibility, autonomy in the profession, and time required to qualify for the profession [58]. It has been suggested that “a mismatch between expectations of a professional role and career, and the reality experienced by those in that role and career, may have implications for retention in the profession” and contribute to nurses leaving their work ([58], p. 396).

Interestingly, the results of the present survey contrast with preexisting research suggesting that financial remuneration is one of the largest motivators for nurses to work in LTC [59], which is notably missing as a top motivator. Despite this, respondents who reported they left work in LTC and HCC did report “better pay elsewhere” as one of their top reasons for leaving the sectors. The idea that “not feeling valued” was a top reason for leaving work in both LTC and HCC speaks to the workplace culture within these sectors for healthcare personnel and prompts further research investigating what it means for a nurse to be “valued” within LTC and HCC. Understanding what prompts a feeling of “value” for staff within these sectors could highlight ways to improve the sense of “value” apart from traditional monetary incentives.

With respect to “work–life balance”—defined as “tackling the problem of increasing amounts of stress in the workplace as people try to juggle a wide range of factors in their life/work environment, including work; family; friends; health; and spirit/self” ([60], p. 53)—previous research has suggested that nursing leaders are challenged to meet the organizational demands in the LTC environment that is “overwhelmingly stressful,” resulting in poor work–life balance ([61], p. 405 [62]). Kelly et al. [61] suggest that the demands nurses are challenged to meet include “managing multiple, complex and competing priorities, such as workforce engagement, clinical outcomes, and fiscal productivity” (p. 404). In the present study, results suggest that struggling to meet these demands may contribute to poor retention of new nurses in the sector [59, 63].

Nurses’ beliefs about LTC, HCC, and hospital sectors may be strongly related to retention within these sectors [59]. The findings of the present study align with the results of a systematic review investigating the factors that influence retention among hospital nurses [59]. Nine domains increasing staff turnover were found in their review: nursing leadership and management (e.g., a lack of support from managers), education and career advancement (e.g., lack of professional development opportunities), organizational work environment (e.g., poor access to resources like Personal Protective Equipment), staffing levels (e.g., high patient‐to‐nurse ratios), professional issues (e.g., out‐of‐date nursing skills, low autonomy), support at work (e.g., cynicism), personal influences (e.g., maintaining work–life balance), financial remuneration (e.g., unsatisfactory salary and benefits) in addition to some demographic influences (e.g., older age and nearing retirement). In the present survey, with respect to both LTC and HCC, respondents agreed least that there were sufficient staffing and resources and that the pay was fair. If the beliefs of healthcare personnel are tied to retention in LTC and HCC, these findings raise concern and point to the need for enhanced support in staffing, management, resources, and financial remuneration. Future research should further investigate the potential relationship between beliefs about staffing and resources, actual staffing and resource availability, and retention within different LTC and HCC employments.

### 5.1. Limitations

As a cross‐sectional survey, these results present data from a single point in time across one province in Canada. Notably, these data were collected in the wake of the COVID‐19 pandemic. This context is important to understanding the experiences of RPNs and RNs within LTC, HCC, and hospital sectors during a time with great potential for higher levels of burnout [32, 33, 35] and exacerbated staffing shortages [34]. Convenience sampling was also used for this survey, with a broad provincial‐wide recruitment strategy. As a result, our findings are not generalizable to all nurses.

## 6. Conclusion

Nurses may leave work in LTC and/or HCC because they are seeking better work–life balance, an increase in pay, and because they do not feel valued or appreciated by their employer. While nurses agree that their schooling has prepared them well to work in LTC and HCC, they also agree that they would want to work within these sectors if they were able to determine their own availability. Employers and educators should leverage healthcare professionals’ reported beliefs to ensure their expectations of work within these sectors are aligned. This could decrease nurse turnover and improve retention, especially in sectors like LTC and HCC. Future research should consider investigating the self‐reported motivators respondents indicated would prompt them to work in LTC/HCC, including what facilitates a feeling of being “valued” in these sectors outside of financial components limited by ministry and funding guidelines. Further, examination of the views of personal support workers, who comprise much of the unregulated workforce in sectors that care primarily for older adults, like LTC and HCC, should be included in future research.

## Author Contributions

D.M.C.: conceptualization, methodology, data analysis, funding acquisition, and writing–review and editing. N.A.G.: conceptualization, data curation, writing–original draft, review and editing, formal analysis, methodology, and project administration. K.P.: conceptualization, methodology, formal analysis, and writing–review and editing. M.E.H.: conceptualization, methodology, formal analysis, and writing–review and editing.

## Funding

This research grant was awarded to Dr. Denise M. Connelly by the Registered Practical Nurses Association of Ontario (WeRPN) Bridging Educational Grant in Nursing (BEGIN) Program funded by the Ontario Ministry of Health and Ministry of Long‐Term Care.

## Conflicts of Interest

The authors declare a conflict of interest whereby the funders of this project assisted with the recruitment of participants. The authors declare no other conflicts of interest.

## Data Availability

The data that support the findings of this study are available upon request from Dr. Denise M. Connelly (corresponding author). The data are not publicly available due to ethical restrictions.
